# Study of the Royal Jelly Free Fatty Acids by Liquid Chromatography-High Resolution Mass Spectrometry (LC-HRMS)

**DOI:** 10.3390/metabo10010040

**Published:** 2020-01-16

**Authors:** Maroula G. Kokotou, Christiana Mantzourani, Rodalia Babaiti, George Kokotos

**Affiliations:** Department of Chemistry, National and Kapodistrian University of Athens, 15771 Athens, Greece; mkokotou@chem.uoa.gr (M.G.K.); chrmantz@chem.uoa.gr (C.M.); rbampait@chem.uoa.gr (R.B.)

**Keywords:** 10-Hydroxy-2-decenoic acid, fatty acids, high resolution mass spectrometry, royal jelly

## Abstract

The lipidome of royal jelly (RJ) consists of medium-chained (8–12 carbon atoms) free fatty acids. We present herein a liquid chromatography-high resolution mass spectrometry (HRMS) method that permits the determination of RJ fatty acids and at the same time the detection of suspect fatty acids. The method allows for the direct quantification of seven free fatty acids of RJ, avoiding any derivatization step. It was validated and applied in seven RJ samples, where the major RJ fatty acid trans-10-hydroxy-2-decenoic acid (10-HDA) was found to vary from 0.771 ± 0.08 to 0.928 ± 0.04 g/100 g fresh RJ. Four additional suspect fatty acids were simultaneously detected taking advantage of the HRMS detection.

## 1. Introduction

Royal jelly (RJ), a secretion from the hypopharyngeal and mandibular glands of worker bees, has been used since ancient times in traditional medicine, and it is currently used as a functional food and in the pharmaceutical and cosmetic fields. It is a white-yellowish, gelatinous, acidic colloid, which contains 3% to 8% lipids [1]. In contrast to fatty acids of most animal and plant materials, which consist of 14–20 carbon atoms in the form of esterified triacylglycerols, RJ fatty acids are medium-chained (8–12 carbon atoms) free fatty acids, either terminally and/or internally hydroxylated, or containing a second terminal carboxylic acid functionality, saturated or monounsaturated at the 2-position [1]. The predominant fatty acid in RJ is trans-10-hydroxy-2-decenoic acid (10-HDA), also known as queen bee acid, and the amount of this unique fatty acid in pure RJ varies depending on the origin of the jelly and the characteristics of the bee.

RJ is an important functional food because of its human health promoting properties. As summarized in various reviews [1,2,3,4], it has been demonstrated to possess a variety of bioactive properties such as antibacterial, immunomodulatory, wound-healing, growth promoting, vasodilative and hypotensive, anti-inflammatory and antitumor activities. Many of the biological properties of RJ are attributed to its unusual bioactive fatty acid components, in particular to 10-HDA. Melliou and Chinou extensively studied RJ, isolated and characterized new fatty acids from dichloromethane and methanol extracts and demonstrated interesting antimicrobial activities [5]. Watanabe et al. showed that the major specific hydroxy fatty acids (10-HDA and 10-hydroxydecanoic acid) of RJ activate transient receptor potential ankyrin 1 (TRPA1) receptor and such an activation induces thermogenesis and energy expenditure [6]. 10-HDA was found to inhibit LPS-induced IL-6 production and NF-κB activation in a dose-dependent manner [7], and to exert immunomodulatory effects on human monocyte-derived dendritic cells [8]. More recently, the in vitro anti-inflammatory effects of these RJ fatty acids as well as sebacic acid (decanedioic acid) was evaluated and compared in lipopolysaccharide-stimulated RAW264.7 macrophages [9], while the bactericide and anti-inflammatory activity of 10-HDA in human colon cancer cells was studied [10]. Another interesting report demonstrated that 10-HDA promotes the growth and protection of neurons, reduces anxiety-like phenotypes, and benefits bone, muscle and adipose tissues in a sex-dependent manner [11].

Early reports focused their attention on the determination of the major fatty component 10-HDA acid in RJ and RJ products describing the use of liquid chromatography methods [12,13,14]. Later on, comparison between HPLC and UPLC methods as well as capillary zone electrophoresis (CZE) and HPLC methods for the determination of 10-HDA have been published [15,16]. Isidorov et al. characterized various organic acids as well as other volatile components of RJ extracts by gas chromatography-mass spectrometry (GC-MS) [17,18]. More recently, Ferioli et al. compared the lipid content, fatty acid profile and sterol composition in local Italian and commercial RJ samples employing GC-MS [19]. However, in the GC-MS methods the conversion of free fatty acids into the corresponding trimethylsilyl derivatives is required. Most recently, Yamaga et al. studied the metabolism and pharmacokinetics of the RJ fatty acids after oral administration of RJ tablets to healthy subjects using LC-MS/MS [20].

The aim of our work was to develop a method for the study of fatty acids present in RJ using liquid chromatography coupled to high resolution mass spectrometry (LC-HRMS). A convenient and effective extraction protocol of RJ fatty acids was followed and the rapid determination of free fatty acids in RJ samples avoiding any derivatization step was achieved. Employing HRMS offers the possibility to simultaneously screen for additional suspect free fatty acids that might be present in RJ samples, without the need of reference compounds.

## 2. Results and Discussion

### 2.1. ESI-MS/MS Data

Three hydroxy RJ fatty acids (the most abundant 10-HDA, 10-hydroxydecanoic acid and 3-hydroxydecanoic acid) and two dicarboxylic RJ fatty acids (decanedioic acid and 2-dodecenedioic acid) were studied, together with two non-functionalized usual fatty acids of similar chain length (decanoic acid and dodecanoic acid). The high-resolution mass spectra of the compounds studied were recorded in electrospray ionization (ESI) negative mode and are presented in the Supplementary Materials (Figure S1). The structures of the compounds and the exact masses of the deprotonated molecular ions (theoretical and measured) are summarized in Table 1. The most abundant peak in full scan ESI-HRMS spectra was the deprotonated molecular ion [M − H]^−^ for all the seven compounds. The MS/MS spectra of the seven analytes in negative ESI mode are also presented in the Supplementary Materials (Figure S2).

The chemical formulas were also identified using the Formula Finder tab from Peak View 2.1. The calculation of isotopic patterns from molecular formulas is essential to understand MS measurements and confirm proposed structures. The simulated tuples of isotopologue masses and probabilities are required for restrictions during unknown identification [21]. The MS Rank values as well as the RDB values for all the elemental compositions of the compounds and their fragments are presented in Table 1. The MS Rank order is based on the MS data using a combination of mass accuracy and match to the theoretical isotope pattern. The RDB value is a formal calculation of the sum of the number of rings and double bonds present in the formula.

**Figure 1:**
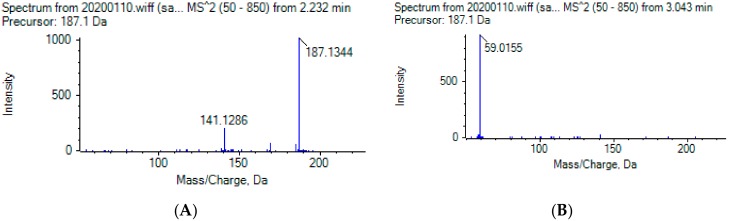

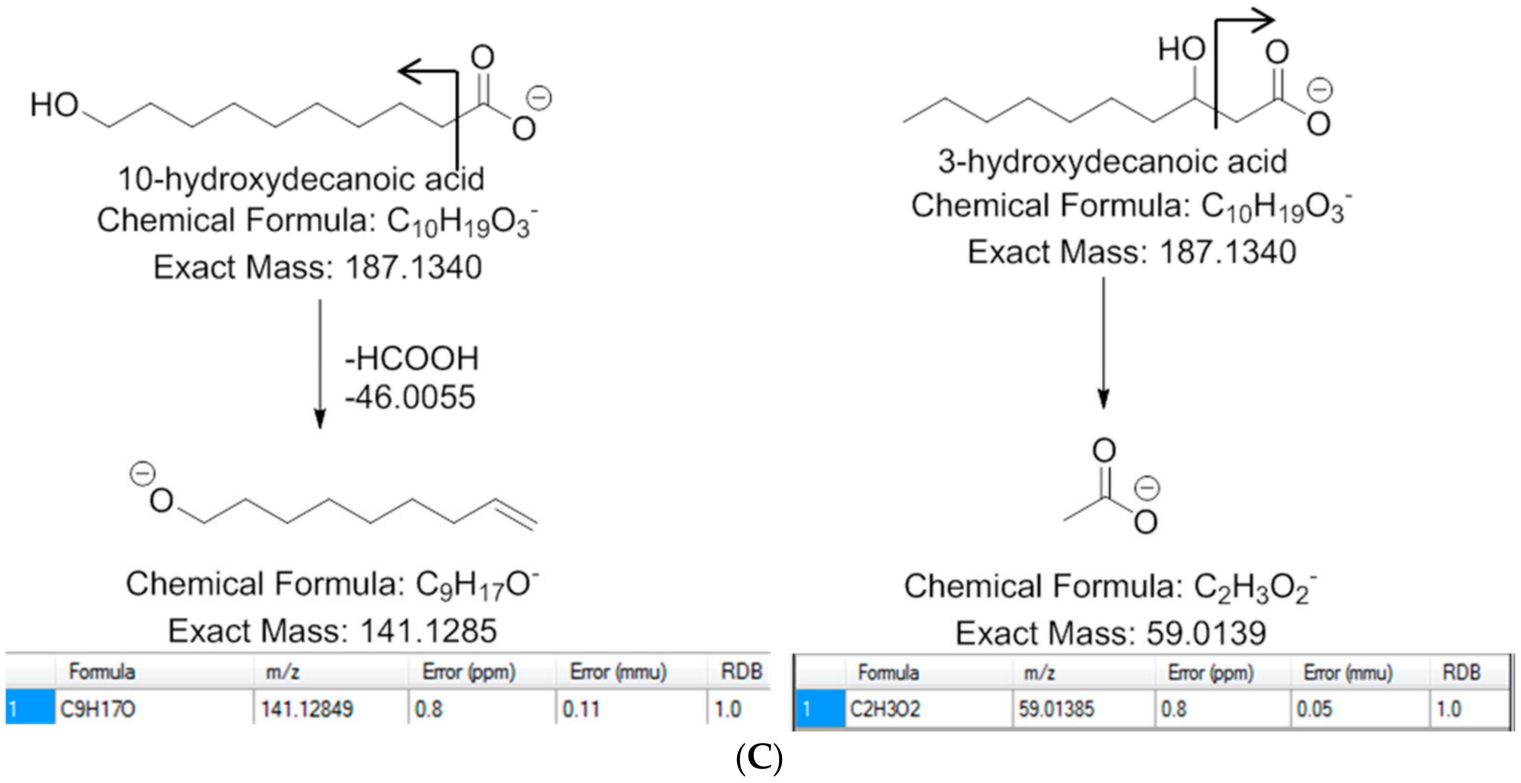
MS/MS spectra of (**A**) 10-hydroxydecanoic acid; (**B**) 3-hydroxydecanoic acid; and (**C**) proposed fragmentation pathways in negative ESI mode.

The isobaric compounds 10-hydroxydecanoic acid and 3-hydroxydecanoic acid can be discriminated by the MS/MS spectra of the precursor ions [M − H]^−^, which are presented in Figure 1. The proposed fragmentation pathways in negative ESI mode are also depicted in Figure 1. In the MS/MS spectrum, 10-hydroxydecanoic acid produces a main fragment at *m*/*z* 141.1286 (Figure 1A), which can be attributed to an α-cleavage resulting to a loss of HCOOH (Figure 1C). In contrast, the most intense ion in the MS/MS spectrum of 3-hydroxydecanoic acid appears at *m*/*z* 59.0155 (Figure 1B), which may result from the generation of CH_3_COO^−^ after β-cleavage (Figure 1C). The isotopic distribution for fragment ions with *m*/*z* 141.1286 and 59.0155 is shown in the Supplementary Materials (Figure S3).

### 2.2. Sample Preparation

Extraction of fatty acids from viscous materials as RJ samples often possesses technical difficulties. In addition, the lipid fraction of RJ consists of unusual medium chain fatty acids containing polar groups. Each one of the fatty acids (10-HDA, 10-hydroxydecanoic acid, 3-hydroxydecanoic acid, decanedioic acid and 2-dodecenedioic acid) contains two functional groups, either a carboxyl and a hydroxyl functionality or two carboxyl functionalities, giving a quite polar character to these fatty acids. Thus, we studied two extraction protocols, one employing methanol and the other a mixture of diethyl ether-isopropanol (50:1 *v*/*v*), as suggested by Ferioli [19].

### 2.3. Method Validation

As summarized in Table 2, excellent linearities between the analyte peak area (y) and the corresponding concentration (x) were obtained. Instrumental limits of detection (LODs) and quantification limits (LOQs) are also presented in Table 2.

Three RJ samples were spiked at 0.5 mg/L of the analytes, and trueness (recovery), precision (repeatability) and the matrix effect were estimated. Better recoveries (82% to 104%) were achieved with the protocol B (Supplementary Materials, Table S1) and the RSD (%) values varied from 2.49% to 13.87%. For 10-HDA and 3-hydroxydecanoic acid, the matrix factor value was <1, which suggests signal suppression in the samples, while for 10-hydroxydecanoic acid, decanedioic acid, 2-dodecenoic acid, decanoic acid and dodecanoic acid, the matrix factor value was >1, which denotes signal enhancement (Supplementary Materials, Table S1). The diethyl ether-isopropanol (50:1 *v*/*v*) protocol was applied for the analysis of real samples.

### 2.4. Analysis of RJ Samples

**Figure 2:**
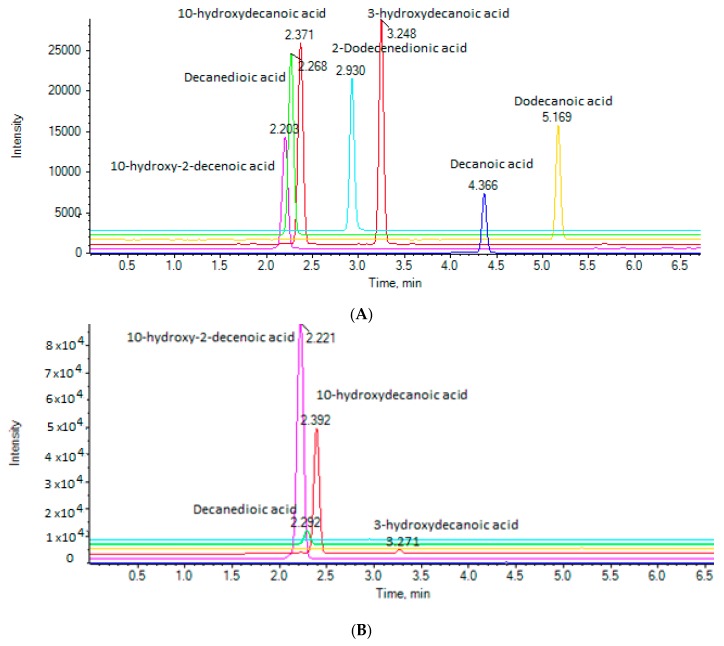
Extracted ion chromatograms (EICs) of the analytes in (**A**) a standard solution (500 ng/mL); and (**B**) a RJ sample (500-fold dilution).

Seven different RJ samples, which were purchased from the local market, were analyzed. The extracted ion chromatograms (EICs) of the analytes in a standard solution (A), as well as in a representative RJ sample (B) are presented in Figure 2. The isobaric 10-hydroxydecanoic acid and 3-hydroxydecanoic acid are well separated and are eluted at 2.37 and 3.25 min, respectively.

**Figure 3:**
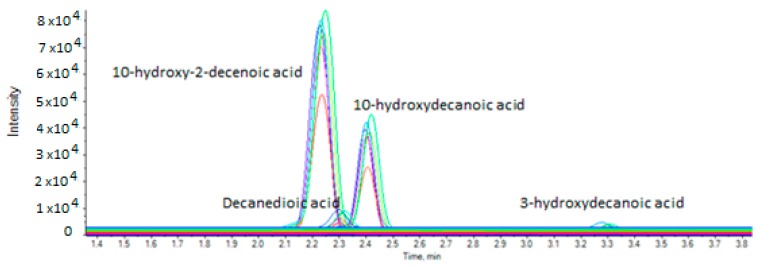
Multi sample analysis (seven RJ samples).

The concentrations of the seven analytes in seven RJ extracts (in triplicate) are summarized in Table 3. Figure 3 represents the multi sample analysis data. The contents of free fatty acids are expressed as g of fatty acid per 100 g of fresh RJ. 10-HDA was found to be the most abundant, as expected, and its content ranged from 0.771 ± 0.08 to 0.928 ± 0.04 g/100 g fresh RJ. These results are in accordance with literature taking into account that the content of 10-HDA depends on the origin of the jelly and the characteristics of the bee. The levels of 10-HDA have been found to vary from 0.75 to 2.54 by Genc and Aslan using column liquid chromatography [13], and from 0.8 to 3.2 by Ferioli et al. using either CZE or HPLC [16].

Among the other fatty acids, 10-hydroxydecanoic acid was present at considerably higher concentrations than the rest and its content varied from 0.285 ± 0.03 to 0.366 ± 0.02 g/100 g of fresh RJ. 3-Hydroxydecanoic acid content accounts on approximately one tenth of 10-hydroxydecanoic acid (0.028 ± 0.003 to 0.036 ± 0.001 g/100 g of fresh RJ). Both dicarboxylic acids decanedioic and 2-dodecenedioic were found at low concentrations varying from 0.075 ± 0.002 to 0.122 ± 0.008 and around 0.012 ± 0.00, respectively. The usual fatty acids of similar chain length, decanoic and dodecanoic, were determined on RJ samples at very low concentrations (from 0.002 ± 0.000 to 0.003 ± 0.000).

Clearly, terminally hydroxylated decanoic acids, either unsaturated at 2-position or saturated, are the major components of the lipidic part of RJ. Dicarboxylic acids of 10 or 12 carbon atoms, saturated or unsaturated, are present in considerably lower amounts than the hydroxylated fatty acids, while the native fatty acids of similar chain length widely distributed in other natural products are found only at very low concentrations in RJ.

### 2.5. Suspect Analysis

In recent years, non-targeted as well as suspect screening approaches have increasingly attracted the interest for the tentative identification of unknown or suspect components in foods [22,23]. Accurate mass measurement and characteristic fragmentation are powerful tools for the elucidation of the molecular structure of unknown and suspect compounds. Thus, in addition to targeted analysis using reference standards, HRMS detection offers the possibility to simultaneously screen for additional suspect species, in particular free fatty acids that might be present in RJ samples, without the need of reference compounds. The existence of four such fatty acids previously reported as RJ components, which are not commercially available, namely 8-hydroxyoctanoic acid, 3-hydroxyoctanoic acid, 3,10-dihydroxydecanoic acid, and 2-decenedioic acid, was explored in our RJ samples by LC-HRMS.

In suspect screening, the masses of the deprotonated ions were calculated on the basis of the molecular formula, and EICs were created in MultiQuant 3.0.2 (Supplementary Materials, Figure S4). All four fatty acids were tentatively identified in RJ samples with good ion intensities and peak areas. The suspect analysis data are summarized in Table 4. The results showed high mass accuracy (less than 5 ppm) and acceptable isotopic fit values (MS Rank). If one or more peaks were detected with the use of EICs, the isotopic pattern and the MS/MS fragments were examined in PeakView 2.1 to confirm that the peak represents the suspect compound. The comparison and interpretation of the MS/MS fragments were performed with use of literature data and spectral libraries such as MassBank [24]. The full scan spectra of 3,10-dihydroxydecanoic acid, 8-hydroxyoctanoic acid, 3-hydroxyoctanoic acid and 2-decenedioic acid as well as their MS/MS spectra are depicted in Supplementary Materials (Figures S5 and S6).

**Figure 4:**
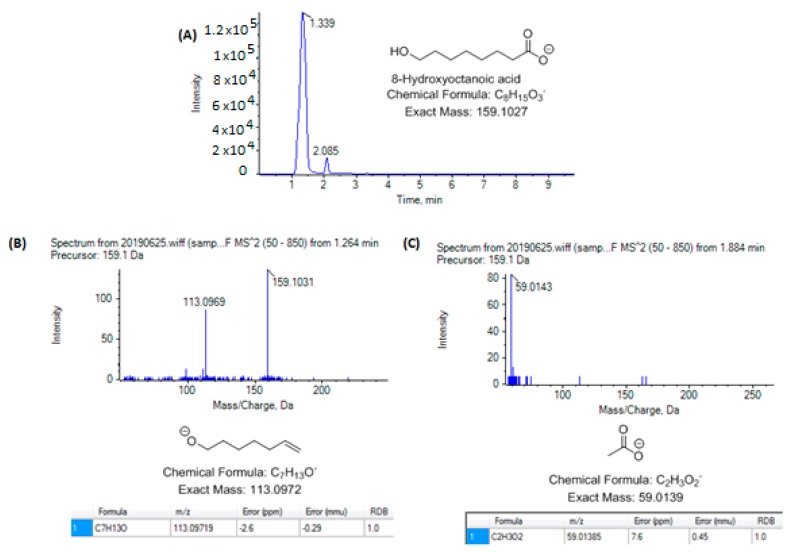
(**A**) EIC corresponding to exact mass of deprotonated hydroxyoctanoic acid; (**B**) MS/MS spectrum of the ion corresponding to peak eluted at 1.34 min; (**C**) MS/MS spectrum of the ion corresponding to peak eluted at 2.09 min.

Two isomers of hydroxyoctanoic acid (molecular formula C_10_H_20_O_4_), one terminally hydroxylated and the other hydroxylated at the 3- position, have been reported to be present in RJ [18]. As in the case of hydroxylated decanoic acids, 8-hydroxyoctanoic acid has been reported to exist in much higher quantity than 3-hydroxyoctanoic acid [18]. The EIC corresponding to exact mass of 159.1027 (deprotonated molecular ion of hydroxyoctanoic acid) (Figure 4A) revealed the existence of two isomeric isobaric fatty acids, the first one eluted at 1.34 min and the second one at 2.09 min. The MS/MS spectra of the precursor ions corresponding to these two peaks are presented in Figure 4B,C. The main fragment in 4B shows a loss of 46.0055 (loss of HCOOH) indicating that the precursor ion may correspond to 8-hydroxyoctanoic acid. Confirmation of this assignment is the comparison of the MS/MS spectrum of 8-hydroxyoctanoic acid with that found in the database Mass Bank [24], showing the same main fragment ion at *m/z* 113.0969. The main peak of the second MS/MS spectrum (Figure 4C) appeared at *m/z* 59.0143, which may correspond to CH_3_COO^−^ after β-cleavage. Unfortunately, the MS/MS spectrum of 3-hydroxyoctanoic acid is not available in the database Mass Bank. The isotopic distribution for fragment ions with *m/z* 113.0969 and 59.0143 is shown in the Supplementary Materials (Figure S7). In analogy to the fragmentation we proposed for 10-hydroxydecanoic and 3-hydroxydecanoic acids (Figure 1), we propose that peak A (Figure 4A) corresponds to 8-hydroxyoctanoic acid, while peak B (Figure 4A) corresponds to 3-hydroxyoctanoic acid. This assignment is in agreement with the observed order of elution for the isobaric 10-hydroxydecanoic and 3-hydroxydecanoic acids (Figure 2).

8-Hydroxyoctanoic acid and 3-hydroxyoctanoic acid were found to be 9% and 0.2%, respectively, relative to 10-HDA (Table 4), as calculated using the areas recorded by LC/HRMS. Another major component of RJ is 3,10-dihydroxydecanoic acid (molecular formula C_10_H_20_O_4_). The EIC for its [M-H]^-^ exact mass (203.1289) showed an area leading to 15% content relative to 10-HDA. Finally, the EIC for the exact mass 199.0976 corresponding to 2-decenedioic acid showed a 5% content relative to 10-HDA. Taking into consideration the variations between RJ samples of different origin for the contents of these four fatty acids, we may conclude that a good estimation of the content of suspect compounds of RJ may be performed by LC/HRMS.

### 2.6. Biological Significance

As described in the introduction, the unusual free fatty acids of RJ have been shown to exhibit a variety of bioactivities such as antimicrobial, anti-inflammatory, immunomodulatory, and growth promoting activities [5,6,7,8,9,10,11]. However, no attention has been paid so far on the potential interactions of RJ fatty acids with free fatty acid receptors. Work during the last two decades has shown that non-esterified medium-chained fatty acids can directly regulate biological processes such as metabolic and immune functions through interactions with free fatty acid receptors, such as GPR40 and GPR84 [25]. Medium-chained fatty acids may activate GPR40 and as a result they may regulate insulin secretion from pancreatic β cells [26,27]. In addition, medium-chained fatty acids, such as decanoic and dodecanoic acids, may act as ligands for orphan G protein-coupled receptor GPR84 [28]. Notably, GPR84 was found to be activated by medium-chained fatty acids possessing a hydroxyl group at the 3-position more effectively than by the corresponding non-hydroxylated acids [29]. Hence, the 3-hydroxy medium-chained fatty acids found in RJ may exert bioactivities through interactions with free fatty acid receptors.

**Figure 5:**
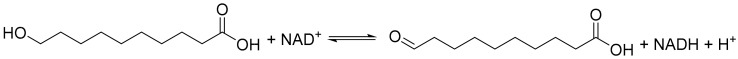
Metabolic pathway of 10-hydroxydecanoic acid [30].

As far as the metabolism of the hydroxylated fatty acids, as reported in Kyoto Encyclopedia of Genes and Genomes (KEGG) [30], 10-hydroxydecanoic acid may be oxidized to the corresponding aldehyde, as shown in Figure 5. It may be assumed that other terminally hydroxylated fatty acids of RJ (for example, trans-10-hydroxy-2-decenoic acid, 8-hydroxyoctanoic acid, 3,10-dihydroxydecanoic acid) may be metabolized in a similar manner.

## 3. Materials and Methods

### 3.1. Chemicals and Reagents

All solvents used were of LC-MS analytical grade. Acetonitrile was purchased from Carlo Erba (Val De Reuil, France), isopropanol and methanol from Fisher Scientific (Loughborough, UK) and formic acid 98% to 100% from Chem-Lab (Zedelgem, Belgium). 10-Hydroxydecanoic acid was purchased from Fluorochem (Derbyshire, UK) and was of the highest available quality. 10-HDA, 3-hydroxydecanoic acid, decanedioic acid and 2-dodecenedioic acid were synthesized in the Laboratory of Organic Chemistry (National and Kapodistrian University of Athens). All physical data were in agreement with literature data and their purity was >97%.

### 3.2. Stock and Working Solutions

Stock solutions of the standard compounds were prepared in a concentration of 1000 mg/L in methanol and stored at 4 °C. Working standard solutions were prepared daily by appropriate dilution. The stability of standard solution of the compounds (500 μg/L) was examined within 30 days at 4 °C. The retention times and the peak areas of the analytes were not changed for the freshly prepared standard solutions that were kept in the fridge.

### 3.3. Instrumentation

LC-MS/MS measurements were performed with an ABSciex Triple TOF 4600 combined with a micro-LC Eksigent and an autosampler set at 5 °C and a thermostatted column compartment. Electrospray ionization (ESI) in negative mode was used for the MS experiments. The data acquisition method consisted of a TOF-MS full scan *m/z* 50–850 Da and several IDA-TOF-MS/MS (Information Dependent Acquisition) product ion scans using 40 V Collision Energy (CE) with 15 V (Collision Energy Spread) CES used for each candidate ion in each data acquisition cycle (1091). This workflow allows quantitation (using TOF-MS primarily) and confirmation (using IDA-TOF-MS/MS) in a single run. Halo C18 2.7 μm, 90 Å, 0.5 × 50 mm^2^ from Eksigent was used as a column and the mobile phase consisted of a gradient (A: acetonitrile/0.01% formic acid/isopropanol 80/20 *v*/*v*; B: H_2_O/0.01% formic acid). The elution gradient adopted started with 5% of phase B for 0.5 min, gradually increasing to 98% in the next 7.5 min. These conditions were kept constant for 0.5 min, and then the initial conditions (95% solvent B, 5% solvent A) were restored within 0.1 min to re-equilibrate the column for 1.5 min for the next injection (flow rate 55 µL/min). The data acquisition was carried out with MultiQuant 3.0.2 and PeakView 2.1 from AB SCIEX.

### 3.4. Sample Preparation

Two solid-liquid extraction protocols were studied. For protocol A, an amount of 0.1 g RJ was weighed in a screwcap glass centrifuge tube and methanol (0.3 mL) was added. The sample was stirred for about 30 s and then centrifuged at 4000 rpm for 5 min. The solvent was removed under argon and the residue was reconstituted in 0.1 mL of methanol. LC-MS/MS analysis followed, as described above.

For protocol B, the procedure previously suggested [19] was followed. An amount of 0.1 g RJ was weighed, and the lipid extraction was performed at room temperature by adding 3 mL of diethyl ether/isopropanol 50/1 (*v*/*v*), stirring for about 30 s every 10 min in an overall period of 30 min and then centrifuged at 4000 rpm for 5 min. The supernatant was collected in a glass tube and the extraction procedure was repeated twice more. The combined organic extracts were dried over anhydrous Na_2_SO_4_, and after filtration, the solvent was removed under argon. The dry residue was dissolved in 1 mL of methanol. For the quantification of highly concentrated components, a 500-fold dilution with methanol/water (50/50, *v*/*v*) was carried out, while a 100-fold dilution was performed for the quantification of the other components. LC-MS/MS analysis followed, as described above.

### 3.5. Method Validation

The linearity and limits of detection (LOD) and quantification (LOQ) were assessed. Solutions from 10 to 2000 ng/mL of 10-HDA, 10-hydroxydecanoic acid, 3-hydroxydecanoic acid, decanedioic acid and 2-dodecenedioic acid [3 replicates; 16 levels (10, 30, 50, 80, 100, 200, 300, 400, 500, 600, 700, 800, 1000, 1300, 1500, 2000 ng/mL); *n* = 3 × 16] and solutions from 5–500 ng/mL of decanoic acid and dodecanoic acid were used for the construction of the linear curves [3 replicates; 8 levels (10, 30, 50, 100, 200, 300, 400, 500 ng/mL); *n* = 3 × 8]. The instrumental detection limits were estimated (LOD = 3.3 * Sa/b, where Sa is the standard deviation of the response of six replicates of the lowest standard and b is the slope of the calibration curve).

Then, RJ samples were spiked at a final concentration of 500 ng/mL (*n* = 3 × 2 = 6) for the recovery, inter-day precision and matrix effect studies. The matrix factor was calculated as the ratio of the peak response in the presence of a matrix to the peak response in the pure solvent.

### 3.6. Royal Jelly Samples

Seven brand products of RJ were collected from the local market in Athens, Greece. Five of them were Greek products, one was a French product and the last one a Spanish product.

## 4. Conclusions

The first LC/HRMS method for the analysis of free fatty acids in RJ samples was developed and validated. A simple solid-liquid extraction protocol was used for the extraction of RJ free fatty acids from the samples exhibiting very good recoveries. Our method is fast and allows for the direct quantification of free fatty acids of RJ, namely 10-HDA, 10-hydroxydecanoic acid, 3-hydroxydecanoic acid, decanedioic acid and 2-dodecenedioic acid, together with usual fatty acids of similar length (decanoic and dodecanoic acids) avoiding any derivatization step. In seven RJ samples, the major RJ fatty acid, 10-HDA, was found to vary from 0.771 ± 0.08 to 0.928 ± 0.04 g/100 g fresh RJ, while the second most abundant fatty acid was 10-hydroxydecanoic acid at concentrations varying from 0.285 ± 0.03 to 0.366 ± 0.02 g/100 g of fresh RJ. In addition, HRMS detection offers the advantage to simultaneously screen for additional suspect fatty acids that might be present in RJ samples, without the need of reference compounds.

## Figures and Tables

**Table 1:** Fatty acids used in the chromatographic method and mass spectral data.

Compound	Structure	Theoretical Mass [M − H]^−^	Measured Mass [M − H]^−^	Elemental Composition	MS Rank ^a^	RDB ^b^
10-HDA	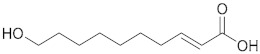	185.1183	185.1181 MS/MS 139.1122	C_10_H_17_O_3_^−^ C_9_H_15_O^−^	1/1 1/1	2 2
10-Hydroxydecanoic acid	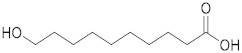	187.1340	187.1335 MS/MS 141.1286	C_10_H_19_O_3_^−^ C_9_H_17_O^−^	1/1 1/1	1 1
3-Hydroxydecanoic acid	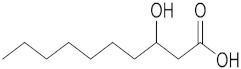	187.1340	187.1341 MS/MS 59.0155	C_10_H_19_O_3_^−^ C_2_H_3_O_2_^−^	1/1 1/1	1 1
Decanedioic acid	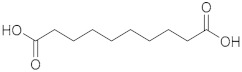	201.1132	201.1125 MS/MS 139.1133 183.1030	C_10_H_17_O_4_^−^ C_9_H_15_O^−^ C_10_H_15_O_3_^−^	1/1 1/1 1/1	2 2 3
2-Dodecenedioic acid	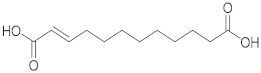	227.1289	227.1284 MS/MS 183.1398	C_12_H_19_O_4_^−^ C_11_H_19_O_2_^−^	1/1 1/1	3 2
Decanoic acid	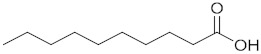	171.1391	171.1388 MS/MS -	C_10_H_19_O_2_^−^ -	1/1 -	1 -
Dodecanoic acid	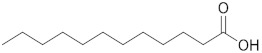	199.1704	199.1703 MS/MS -	C_12_H_23_O_2_^−^ -	1/1 -	1 -

^a^ MS Rank: The rank order based on the MS data. This uses a combination of mass accuracy and match to the theoretical isotope pattern. (1st of 1 hit), ^b^ RDB: The double bond equivalent that is a formal calculation of the sum of the number of rings and double bonds present in the formula.

**Table 2:** Calibration curve data as well as limits of detection (LOD) and quantification (LOQ).

Analyte	Range (ng/mL)	Calibration Equations	Linearity (R^2^)	LOD (ng/mL)	LOQ (ng/mL)
10-HDA	10–2000	y = 70x − 4360	0.993	207	628
10-Hydroxydecanoic Acid	10–2000	y = 90x − 4199	0.990	282	855
3-Hydroxydecanoic Acid	10–2000	y = 96x − 5325	0.995	197	596
Decanedioic Acid	10–2000	y = 74x − 7432	0.992	200	608
2-Dodecenedioic Acid	10–2000	y = 67x − 7466	0.992	266	805
Decanoic Acid	5–500	y = 22x − 637	0.992	24	80
Dodecanoic Acid	5–500	y = 33x − 643	0.991	18	60

**Table 3:** Contents of free fatty acids in real samples (g/100 g fresh RJ).

Analyte	1	2	3	4	5	6	7	Median
10-HDA	0.892 ± 0.02	0.903 ± 0.08	0.876 ± 0.03	0.771 ± 0.08	0.875 ± 0.03	0.928 ± 0.04	0.914 ± 0.04	0.880 ± 0.05
10-Hydroxy decanoic Acid	0.302 ± 0.03	0.366 ± 0.02	0.303 ± 0.02	0.325 ± 0.05	0.285 ± 0.03	0.342 ± 0.05	0.315 ± 0.01	0.320 ± 0.03
3-Hydroxy decanoic Acid	0.030 ± 0.001	0.032 ± 0.001	0.028 ± 0.003	0.032 ± 0.001	0.036 ± 0.001	0.036 ± 0.001	0.035 ± 0.001	0.032 ± 0.00
Decanedioic Acid	0.089 ± 0.006	0.082 ± 0.002	0.080 ± 0.005	0.075 ± 0.002	0.082 ± 0.006	0.091 ± 0.005	0.122 ± 0.008	0.089 ± 0.04
2-Dodecenedioic Acid	0.012 ± 0.000	0.012 ± 0.000	0.012 ± 0.000	0.012 ± 0.000	0.012 ± 0.000	0.012 ± 0.000	0.012 ± 0.000	0.012 ± 0.00
Decanoic Acid	0.002 ± 0.000	0.003 ± 0.000	0.003 ± 0.000	0.003 ± 0.000	0.003 ± 0.000	0.003 ± 0.000	0.003 ± 0.000	0.003 ± 0.00
Dodecanoic Acid	0.003 ± 0.000	0.003 ± 0.000	0.003 ± 0.000	0.003 ± 0.000	0.002 ± 0.000	0.003 ± 0.000	0.003 ± 0.000	0.003 ± 0.00

**Table 4:** Suspect analysis data.

Compound	Structure	Theoretical Mass [M − H]^−^	Measured Mass [M − H]^−^	Elemental Composition	MS Rank ^a^	RDB ^b^	Content Relative to 10-HDA (%) ^c^
3,10-Dihydroxydecanoic acid	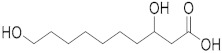	203.1289	203.1286 MS/MS 59.0136	C_10_H_19_O_4_^−^ C_2_H_3_O_2_^−^	1/1 1/1	1 1	15 ± 0.04
8-Hydroxyoctanoic acid	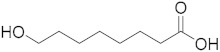	159.1027	159.1024 MS/MS 113.0969	C_8_H_15_O_3_^−^ C_7_H_13_O^−^	1/1 1/1	2 1	9 ± 0.02
3-Hydroxyoctanoic acid	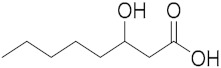	159.1027	159.1024 MS/MS 59.0143	C_8_H_15_O_3_^−^ C_2_H_3_O_2_^−^	1/1 1/1	2 1	0.2 ± 0.00
2-Decenedioic acid	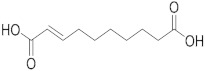	199.0976	199.0979 MS/MS 155.1073	C_10_H_15_O_4_^−^ C_9_H_15_O_2_^−^	1/1 1/1	3 2	5 ± 0.01

^a^ MS Rank: The rank order based on the MS data. This uses a combination of mass accuracy and match to the theoretical isotope pattern. (1st of 1 hit); ^b^ RDB: The double bond equivalent, which is a formal calculation of the sum of the number of rings and double bonds present in the formula; ^c^ By calculation of the area.

## Supplementary Materials

The following are available online at https://www.mdpi.com/2218-1989/10/1/40/s1, Figure S1: Full scan spectra of 10-HDA (A), 10-hydroxydecanoic acid (B), 3-hydroxydecanoic acid (C), decanedioic acid (D), 2-dodecenedioic acid (E), decanoic acid (F) and dodecanoic acid (G), Figure S2: MS/MS spectra of 10-HDA (A), 10-hydroxydecanoic acid (B), 3-hydroxydecanoic acid (C), decanedioic acid (D), 2-dodecenedioic acid (E), decanoic acid (F) and dodecanoic acid (G), Figure S3. Isotopic distribution for fragment ions with *m*/*z* 141.1286 (A) and *m*/*z* 59.0155 (B), Figure S4: EICs of 3,10-dihydroxydecanoic acid (A), 8-hydroxyoctanoic acid and 3-hydroxyoctanoic acid (B) and 2-decenedioic acid (C), Figure S5: Full scan spectra of 3,10-dihydroxydecanoic acid (A), 8-hydroxyoctanoic acid (B), 3-hydroxyoctanoic acid (C) and 2-decenedioic acid (D), Figure S6: MS/MS spectra of 3,10-dihydroxydecanoic acid (A), 8-hydroxyoctanoic acid (B), 3-hydroxyoctanoic acid (C) and 2-decenedioic acid (D), Figure S7. Isotopic distribution for fragment ions with *m*/*z* 113.0969 (A) and *m*/*z* 59.0143 (B), Table S1: Precision and accuracy data in spiked RJ samples.

## References

[1] Ramadana M.F., Al-Ghamdi A. (2012). Bioactive compounds and health-promoting properties of royal jelly: A review. J. Funct. Foods.

[2] Fratini F., Cilia G., Mancini S., Felicioli A. (2016). Royal Jelly: An ancient remedy with remarkable antibacterial properties. Microbiol. Res..

[3] Cornara L., Biagi M., Xiao J., Burlando B. (2017). Therapeutic properties of bioactive compounds from different honeybee products. Front. Pharmacol..

[4] Khazaei M., Ansarian A., Ghanbari E. (2018). New findings on biological actions and clinical applications of royal jelly: A Review. J. Diet. Suppl..

[5] Melliou E., Chinou I. (2005). Chemistry and bioactivity of royal jelly from Greece. J. Agric. Food Chem..

[6] Terada Y., Narukawa M., Watanabe T. (2011). Specific hydroxy fatty acids in royal jelly activate TRPA1. J. Agric. Food Chem..

[7] Sugiyama T., Takahashi K., Tokoro S., Gotou T., Neri P., Mori H. (2011). Inhibitory effect of 10-hydroxy-trans-2-decenoic acid on LPS-induced IL-6 production via reducing IκB-ζ expression. Innate Immun..

[8] Mihajlovic D., Rajkovic I., Chinou I., Colic M. (2013). Dose-dependent immunomodulatory effects of 10-hydroxy-2-decenoic acid on human monocyte-derived dendritic cells. J. Funct. Foods.

[9] Chen Y.F., Wang K., Zhang Y.Z., Zheng Y.F., Hu F.L. (2016). In vitro anti-inflammatory effects of three fatty acids from royal jelly. Mediat. Inflamm..

[10] Yang Y.C., Chou W.M., Widowati D.A., Lin I.P., Peng C.C. (2018). 10-Hydroxy-2-decenoic acid of royal jelly exhibits bactericide and anti-inflammatory activity in human colon cancer cells. BMC Complement. Altern. Med..

[11] Weiser M.J., Grimshaw V., Wynalda K.M., Mohajeri M.H., Butt C.M. (2018). Long-term administration of queen bee acid (QBA) to rodents reduces anxiety-like behavior, promotes neuronal health and improves body composition. Nutrients.

[12] Bloodworth B.C., Harn C.S., Hock C.T. (1995). Liquid chromatographic determination of trans-10-hydroxy-2-decenoic acid content of commercial products containing royal jelly. JAOAC Int..

[13] Genc M., Aslan A. (1999). Determination of trans-10-hydroxy-2-decenoic acid content in pure royal jelly and royal jelly products by column liquid chromatography. J. Chromatogr. A.

[14] Antinellia J.-F., Zeggane S., Davico R., Rognonea C., Faucon J.-P., Lizzani L. (2003). Evaluation of (E)-10-hydroxydec-2-enoic acid as a freshness parameter for royal jelly. Food Chem..

[15] Zhou J., Zhao J., Yuan H., Meng Y., Li Y., Wu L., Xue X. (2007). Comparison of UPLC and HPLC for determination of trans-10-hydroxy-2-decenoic acid content in royal jelly by ultrasound-assisted extraction with internal standard. Chromatographia.

[16] Ferioli F., Marcazzan G.L., Caboni M.F. (2007). Determination of (E)-10-hydroxy-2-decenoic acid content in pure royal jelly: A comparison between a new CZE method and HPLC. J. Sep. Sci..

[17] Isidorov V.A., Czyzewska U., Isidorova A.G., Bakier S. (2009). Gas chromatographic and mass spectrometric characterization of the organic acids extracted from some preparations containing lyophilized royal jelly. J. Chromatogr. B.

[18] Isidorov V.A., Bakier S., Grzech I. (2012). Gas chromatographic–mass spectrometric investigation of volatile and extractable compounds of crude royal jelly. J. Chromatogr. B.

[19] Ferioli F., Armaforte E., Caboni M.F. (2014). Comparison of the lipid content, fatty acid profile and sterol composition in local Italian and commercial royal jelly samples. J. Am. Oil Chem. Soc..

[20] Yamaga M., Tani H., Yamaki A., Tatefuji T., Hashimoto K. (2019). Metabolism and pharmacokinetics of medium chain fatty acids after oral administration of royal jelly to healthy subjects. RSC Adv..

[21] Loss M., Geber C., Corona F., Hollender J., Singer H. (2015). Accelerated isotope fine structure calculation using pruned transition trees. Anal. Chem..

[22] Jia W., Shi L., Chu X. (2018). Untargeted screening of sulfonamides and their metabolites in salmon using liquid chromatography coupled to quadrupole Orbitrap mass spectrometry. Food Chem..

[23] Gauglitz J.M., Aceves C.M., Aksenov A.A., Aleti G., Almaliti J., Bouslimanim A., Brown E.A., Campeau A., Caraballo-Rodríguez A.M., Chaar R. (2020). Untargeted mass spectrometry-based metabolomics approach unveils molecular changes in raw and processed foods and beverages. Food Chem..

[24] Horai H., Arita M., Kanaya S., Nihei Y., Ikeda T., Suwa K., Ojima Y., Tanaka K., Tanaka S., Aoshima K. (2010). MassBank: A public repository for sharing mass spectral data for life sciences. J. Mass Spectrom..

[25] Offermanns S. (2014). Free fatty acid (FFA) and hydroxy carboxylic acid (HCA) receptors. Annu. Rev. Pharmacol. Toxicol..

[26] Briscoe C.P., Tadayyon M., Andrews J.L., Benson W.G., Chambers J.K., Eilert M.M., Ellis C., Elshourbagy N.A., Goetz A.S., Minnick D.T. (2003). The orphan G protein coupled receptor GPR40 is activated by medium and long chain fatty acids. J. Biol. Chem..

[27] Itoh Y., Kawamata Y., Harada M., Kobayashi M., Fujii R., Fukusumi S., Ogi K., Hosoya M., Tanaka Y., Uejima H. (2003). Free fatty acids regulate insulin secretion from pancreatic beta cells through GPR40. Nature.

[28] Wang J., Wu X., Simonavicius N., Tian H., Ling L. (2006). Medium-chain fatty acids as ligands for orphan G protein-coupled receptor GPR84. J. Biol. Chem..

[29] Suzuki M., Takaishi S., Nagasaki M., Onozawa Y., Iino I., Maeda H., Komai T., Oda T. (2013). Medium-chain fatty acid-sensing receptor, GPR84, is a proinflammatory receptor. J. Biol. Chem..

[30] Kanehisa M., Goto S. (2000). KEGG: Kyoto Encyclopedia of Genes and Genomes. Nucleic Acids Res..

