# Supplementary material for: Study of the Royal Jelly Free Fatty Acids by Liquid Chromatography-High Resolution Mass Spectrometry (LC-HRMS)
Source: Metabolites. 2020 Jan 16;10(1):40. doi: 10.3390/metabo10010040 (PMC7022826; doi:10.3390/metabo10010040)
Supplement: Supplementary file 1 [file metabolites-10-00040-s001.pdf]

Study of the Royal Jelly Free Fatty Acids by Liquid Chromatography-High Resolution Mass Spectrometry (LC-HRMS)

Maroula G. Kokotou, Christiana Mantzourani, Rodalia Babaiti and George Kokotos*

Department of Chemistry, National and Kapodistrian University of Athens, Athens 15771, Greece

SUPPLEMENTARY MATERIAL

**Table of Contents**	**Page**
**Figure S1.** Full scan spectra of 10-HDA (A), 10-hydroxydecanoic acid (B), 3-hydroxydecanoic acid (C), decanedioic acid (D), 2-dodecenedioic acid (E), decanoic acid (F) and dodecanoic acid (G).	**S2**
**Figure S2.** MS/MS spectra of 10-HDA (A), 10-hydroxydecanoic acid (B), 3-hydroxydecanoic acid (C), decanedioic acid (D), 2-dodecenedioic acid (E), decanoic acid (F) and dodecanoic acid (G).	**S3**
**Figure S3.** Isotopic distribution for fragment ions with *m/z* 141.1286 (A) and *m/z* 59.0155 (B).	**S4**
**Figure S4.** EICs of 3,10-dihydroxydecanoic acid (A), 8-hydroxyoctanoic acid and 3-hydroxyoctanoic acid (B) and 2-decenedioic acid (C).	**S4**
**Figure S5.** Full scan spectra of 3,10-dihydroxydecanoic acid (A), 8-hydroxyoctanoic acid (B), 3-hydroxyoctanoic acid (C) and 2-decenedioic acid (D).	**S5**
**Figure S6.** MS/MS spectra of 3,10-dihydroxydecanoic acid (A), 8-hydroxyoctanoic acid (B), 3-hydroxyoctanoic acid (C) and 2-decenedioic acid (D).	**S6**
**Figure S7.** Isotopic distribution for fragment ions with *m/z* 113.0969 (A) and *m/z* 59.0143 (B).	**S6**
**Table S1**. Precision and accuracy data in spiked RJ samples.	**S7**

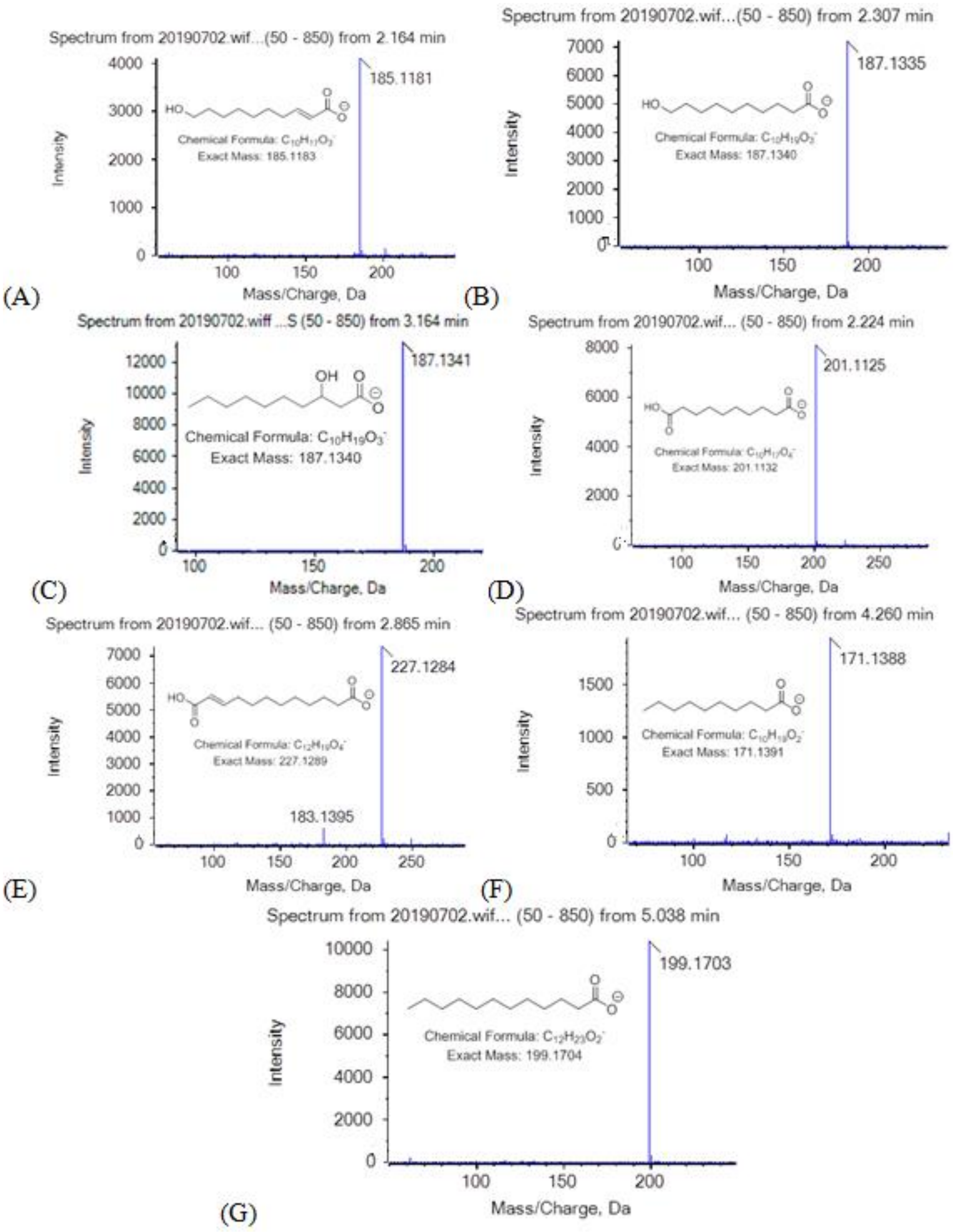

Figure S1. Full scan spectra of 10-HDA (A), 10-hydroxydecanoic acid (B), 3-hydroxydecanoic acid (C), decanedioic acid (D), 2-dodecenedioic acid (E), decanoic acid (F) and dodecanoic acid (G).

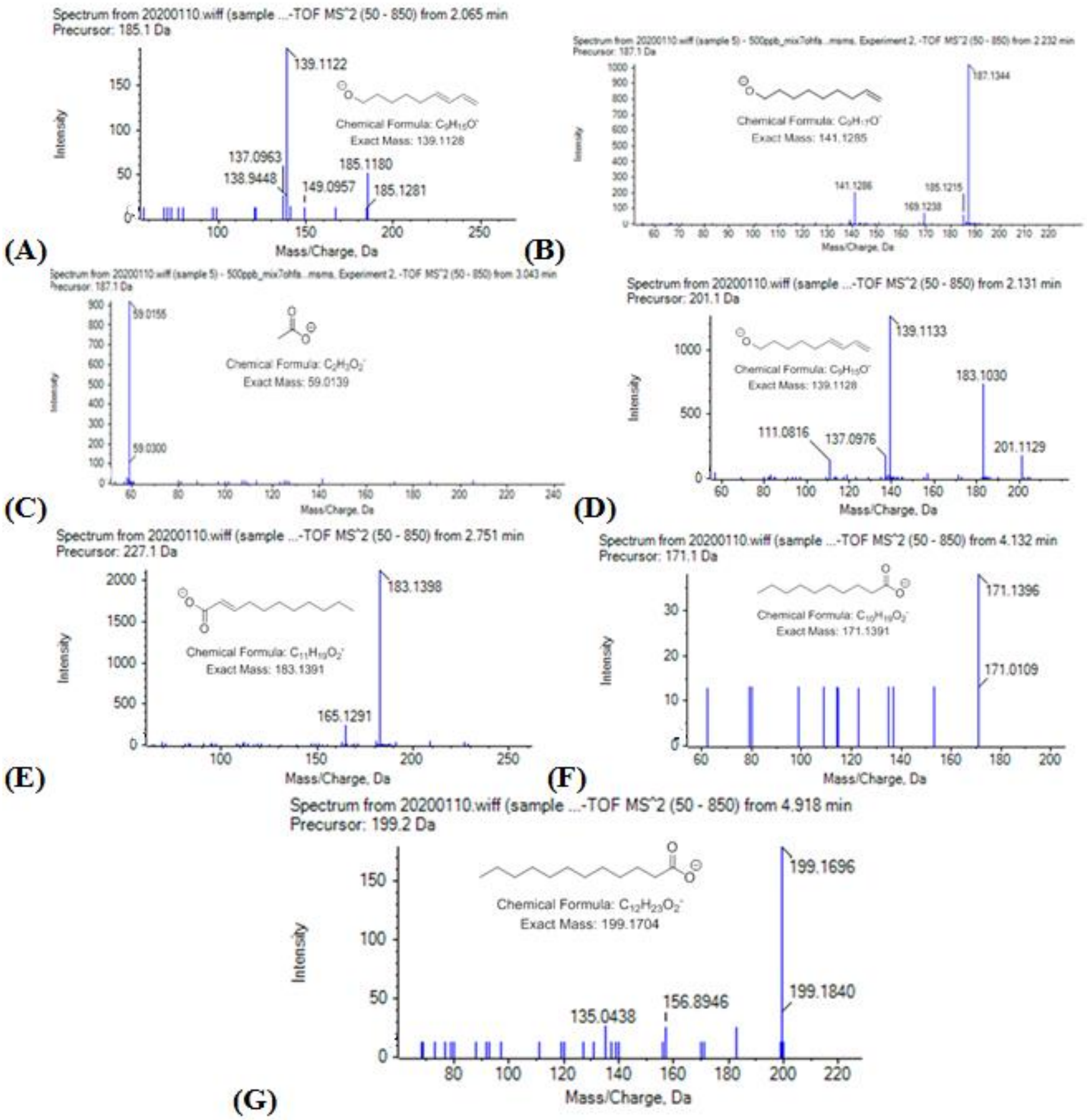

Figure S2. MS/MS spectra of 10-HDA (A), 10-hydroxydecanoic acid (B), 3-hydroxydecanoic acid (C), decanedioic acid (D), 2-dodecenedioic acid (E), decanoic acid (F) and dodecanoic acid (G).

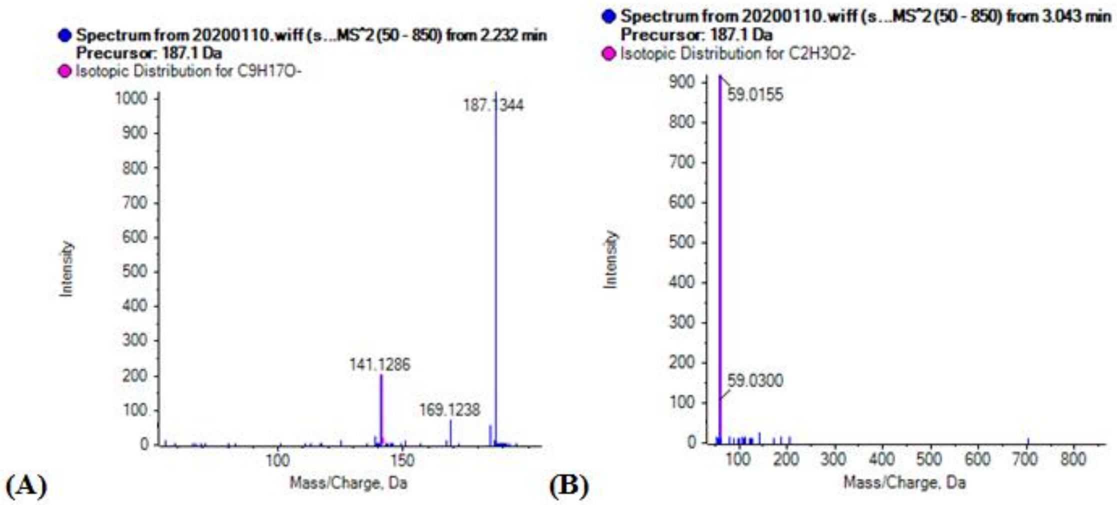

Figure S3. Isotopic distribution for fragment ions with *m/z* 141.1286 (A) and *m/z* 59.0155 (B).

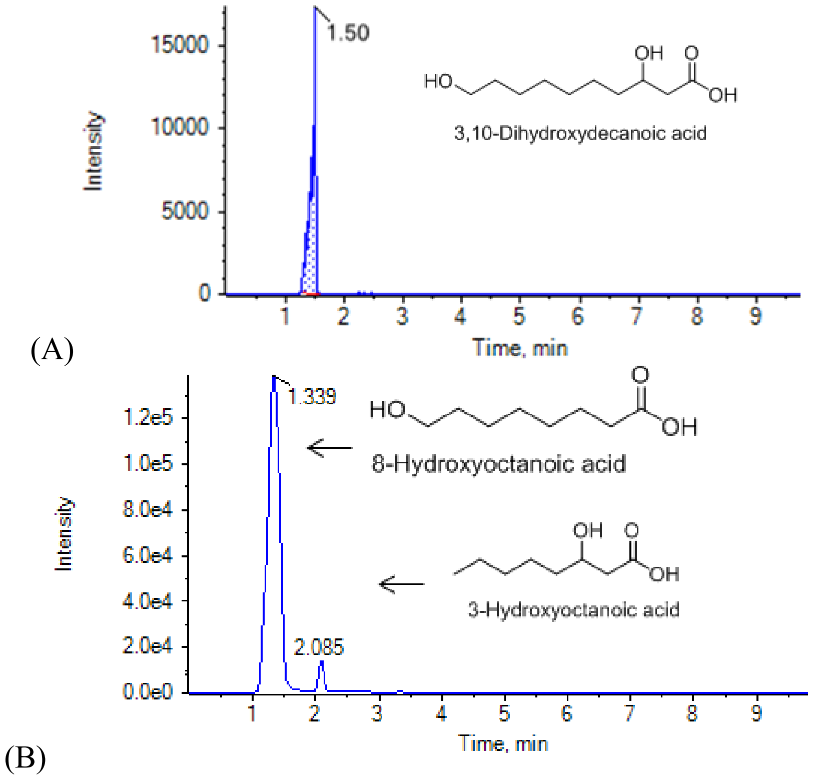

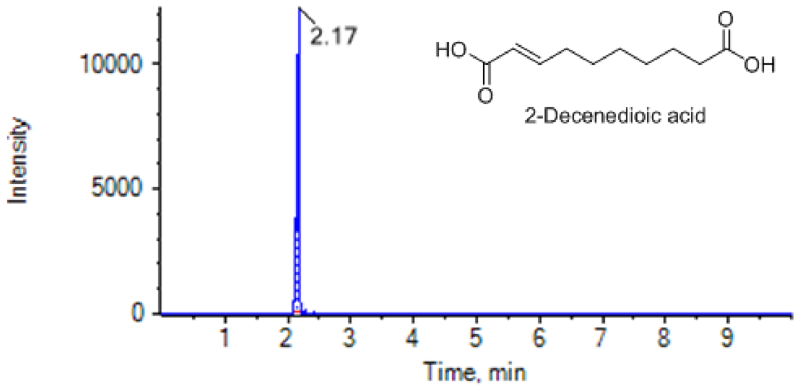

(C)

Figure S4. EICs of 3,10-dihydroxydecanoic acid (A), 8-hydroxyoctanoic acid and 3-hydroxyoctanoic acid (B) and 2-decenedioic acid (C).

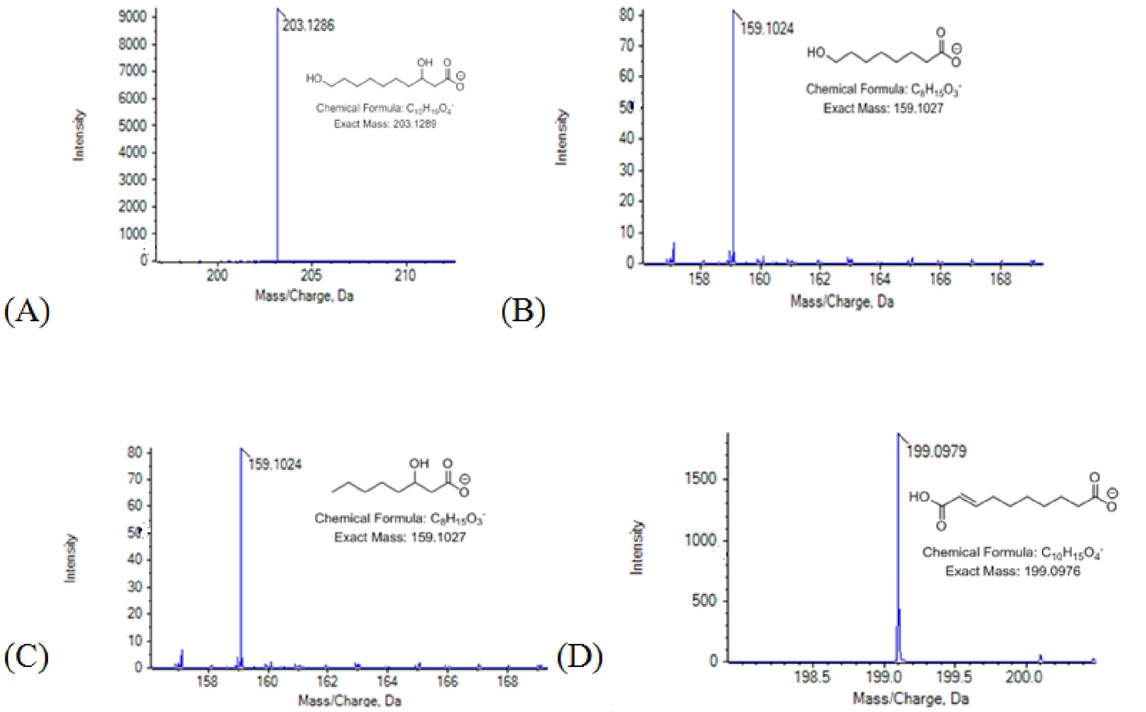

Figure S5. Full scan spectra of 3,10-dihydroxydecanoic acid (A), 8-hydroxyoctanoic acid (B), 3-hydroxyoctanoic acid (C) and 2-decenedioic acid (D).

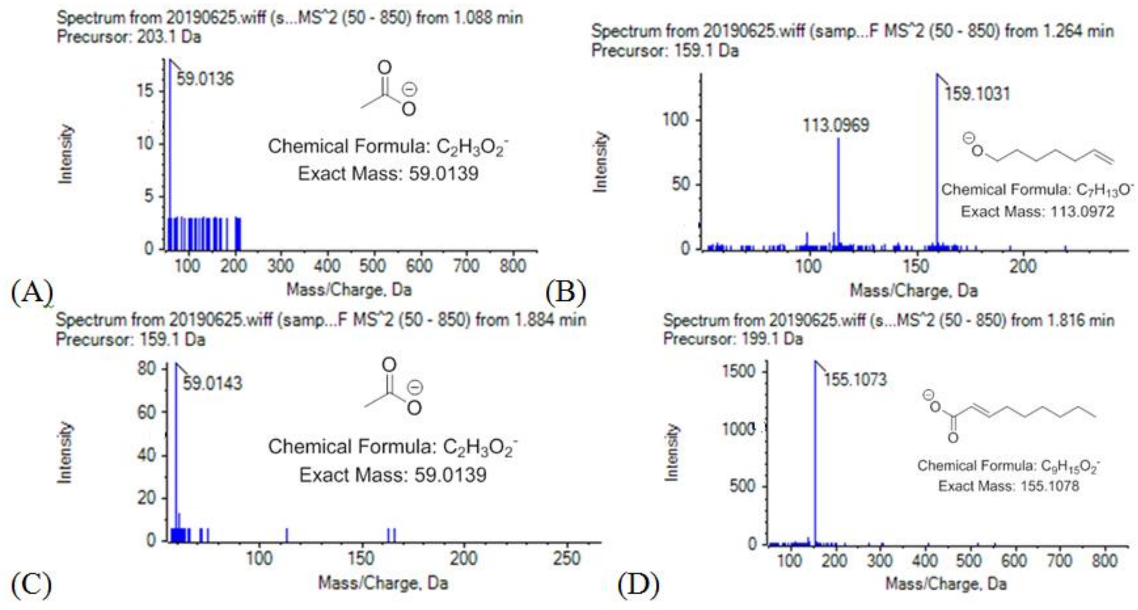

Figure S6. MS/MS spectra of 3,10-dihydroxydecanoic acid (A), 8-hydroxyoctanoic acid (B), 3-hydroxyoctanoic acid (C) and 2-decenedioic acid (D).

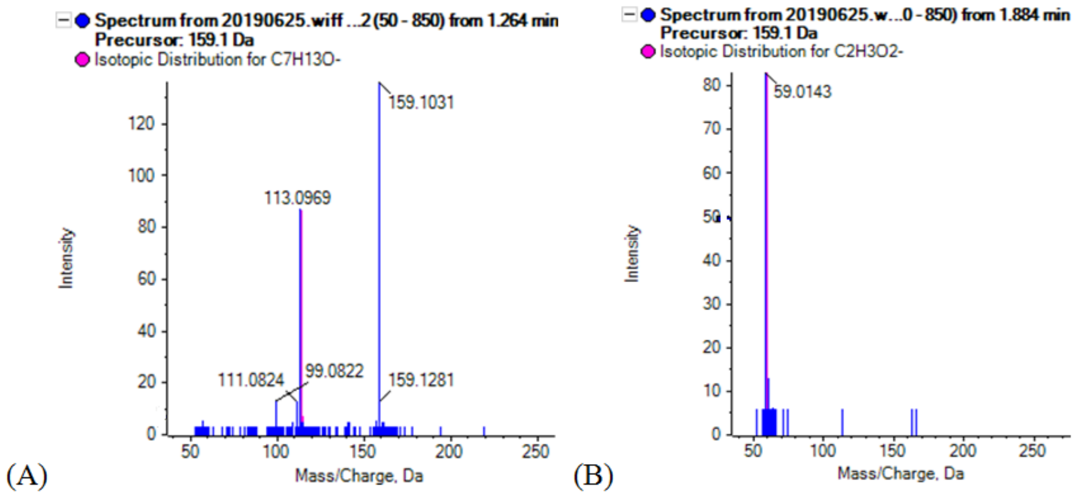

Figure S7. Isotopic distribution for fragment ions with *m/z* 113.0969 (A) and *m/z* 59.0143 (B).

Table S1. Precision and accuracy data in spiked RJ samples.

Compound	Protocol A	Protocol B		
	Recovery (%)	Recovery (%)	RSD (%)	Matrix Factor
10-HDA	82	103	13.87	0.97
10-Hydroxydecanoic acid	113	97	8.79	1.03
3-Hydroxydecanoic acid	81	104	12.80	0.96
Decanedioic acid	85	98	5.63	1.02
2-Dodecenedioic acid	82	97	5.75	1.03
Decanoic acid	-	87	2.49	1.14
Dodecanoic acid	107	82	9.48	1.22

Protocol A was abandoned, because decanoic acid was not recovered.
